# The impact of anal cancer treatment on female sexuality and intimacy: a systematic review

**DOI:** 10.1007/s00520-025-09791-1

**Published:** 2025-08-15

**Authors:** M. Hayes, K. White, R. J. Hillman, C. Rutherford

**Affiliations:** 1 Cancer Care Research Unit, Susan Wakil School of Nursing and Midwifery, The University of Sydney, Sydney, NSW 2006 Australia; 2 St. Vincent’s Hospital, Sydney, Australia; 3 Sydney Local Health District, Sydney, NSW 2050 Australia; 4 The Daffodil Centre, The University of Sydney, a joint venture with Cancer Council New South Wales, Sydney, Sydney, NSW Australia; 5 Sydney Quality of Life Office, Susan Wakil School of Nursing and Midwifery, The University of Sydney, Sydney, NSW 2006 Australia

**Keywords:** Female sexual function, Anal cancer treatment, Radiation therapy, Systematic review

## Abstract

**Purpose:**

Anal cancer has been on the increase for many years. Despite a higher incidence in women, most research focuses on men. Concurrent chemotherapy and radiotherapy is the gold standard of care in the curative setting. While the overall 5-year survival rate is about 70%, many women experience life-altering sequelae of treatment including those affecting sexual function and intimacy. This systematic review aims to identify and synthesise the patient-reported impacts of anal cancer treatment on female sexual function and intimacy.

**Methods:**

We searched five electronic databases: Medline, CINHAL, Scopus, PsycINFO, and Embase from database inception to 7th October 2024. Quantitative and qualitative studies reporting patient-reported sexual function and intimacy impacts following treatment for anal cancer were eligible. Two reviewers independently screened studies and extracted data. We used a narrative synthesis approach to analyse the findings.

**Results:**

Of 338 abstracts retrieved, 26 articles reporting 25 quantitative studies met the eligibility criteria. Findings indicate that overall sexual function was negatively impacted with dyspareunia (pain/discomfort during sex) and sexual function (including interest, arousal, and desire) among the most frequently assessed outcomes. Effects were shown to persist for at least 6-year post-treatment completion resulting in complete abstinence from sexual intercourse for many women.

**Conclusions:**

Concurrent chemotherapy and radiotherapy used in the treatment of anal cancer can have lasting effects on female sexual function and intimacy. The limited available evidence and large amounts of missing data suggest that the severity and extent of this problem are under-reported. Qualitative research may provide a more in-depth understanding.

**Supplementary Information:**

The online version contains supplementary material available at 10.1007/s00520-025-09791-1.

## Introduction

Rates of anal cancer worldwide are increasing and disproportionately affect women [1]. Over 50,000 cases and 19,000 deaths were recorded worldwide in 2020 [2]. In Australia in 2021, 591 new cases were diagnosed, of which 60% were female [3]. High risk human papillomavirus is the primary cause of anal cancer, responsible for up to 96% of cases [4, 5]. Other risk factors include immunosuppression and smoking [6].

Curative treatment for anal cancer has evolved from abdominoperineal resection (APR), which removes the distal colon, rectum, and anal sphincter complex resulting in a permanent colostomy to combined modality therapy following successful clinical trials since the 1970s [7, 8]. Conventional radiotherapy using plain x-ray to facilitate the delivery of radiation to tumour sites was once the preferred curative treatment option [9]. This was superseded by conformal radiotherapy which includes 3D conformal radiotherapy (3D-CRT), intensity modulated radiation therapy (IMRT) and volumetric arc therapy (VMAT). VMAT and IMRT enable precision conformation of the radiation dose to the tumour sites, with minimal doses to surrounding tissues, resulting in fewer toxicities for patients undergoing treatment [10]. The addition of chemotherapy as a radio sensitizer in treatment protocols has further contributed to a reduction in radiation exposure.

With improved survival rates, attention has shifted to the long-term consequences of treatment and the adverse effects on health-related quality of life (HRQL) [11]. Advances in radiation techniques outlined above have reduced overall toxicity to surrounding tissues including genitourinary and gastrointestinal organs. Their specific effects on female sexual function remain varied and complex, leaving it difficult for healthcare professionals to fully understand and manage these side effects of treatment. Disease-associated stigma and shame fuelled by common misconceptions and low levels of awareness about anal cancer can negatively impact HRQL, leading to psychological distress and social isolation. HRQL has been investigated in women with anal cancer, but findings related to sexual function and intimacy are often pooled with data from male participants [12] and/or women with other cancers including cervical and rectal cancer [13, 14]. Without outcome data specific to women with anal cancer, it is inappropriate to assume that treatment effects observed in women with cervical or rectal cancer apply equally to this group.

This systematic review aimed to identify and synthesise evidence on the patient-reported impacts of anal cancer treatment on female sexual function and intimacy, with a focus on 3D-CRT and IMRT radiation modalities. Although 3D-CRT has been superseded by IMRT as the gold standard, many women continue to experience long-term effects from 3D-CRT. Including studies using both modalities was deemed necessary to capture the full scope of patient-reported outcomes.

## Methods

We conducted this review according to the Preferred Reporting Items for Systematic Reviews and Meta-Analysis (PRISMA) statement and PRISMA-S, an extension for reporting literature searches in systematic reviews [15, 16].

### Search strategy

Our search strategy developed with a medical librarian included terms for anal cancer, sexual function, intimacy, and female (Appendix Tables 4, 5, 6, 7, 8, 9, 10, 11). No time or language restrictions were applied. We searched Medline, CINHAL, Scopus, PsycINFO, and Embase from database inception to 7th October 2024. We reviewed reference lists of included studies and four related systematic reviews for additional relevant studies. We contacted conference abstract authors of relevant studies and key researchers in the field.

**Table 1:** Summary of findings from cross-sectional studies (*n* = 16)

First author (year) and country	Study aim	Design, sample size, comparison group, treatment type	PROs assessed (PROM used), time points	Key findings
Bentzen (2013), Norway	To investigate HRQL in the long-term follow-up of anal cancer survivors compared with a reference group from the normal population	*Design:* Retrospective cross-sectional study *Sample:* 101 women median age 61 (40–89) from a total cohort of 128 patients. Scores compared with age- and sex- matched control group *Treatment type:* RT (100%). Technique not disclosed	Sexual function and dyspareunia (EORTC QLQ-CR29^a^) assessed at median 66 m (range 25–112) post-treatment completion	When compared to age- and sex-matched controls, women with anal cancer reported statistically significant worse sexual function and dyspareunia
Corrigan (2022), USA	To use validated PRO questionnaires to survey SCCA survivors about their sexual function more than 2 years after definitive chemoradiation and to identify potential clinical predictors of sexual dysfunction	*Design:* Retrospective cross-sectional study *Sample:* 90 women from a total cohort of 112 patients. Median age of whole sample 65. No comparison group *Treatment type:* Concurrent chemo/IMRT (99%) and RT alone (< 1%)	Sexual function, desire, vaginal symptoms and orgasm function (PROMIS^b^; FSFI^c^) assessed at median 51 m post-treatment completion	Mean overall FSFI scores for 80% of women were below the recommended cut-off of 26.55 indicating sexual dysfunction
Corte (2011), France	To evaluate female sexual function after APR for SCC of the anal canal and to determine whether or not flap reconstruction (intervention) of the vagina improves it	*Design:* Retrospective cross-sectional study *Sample:* 21 women (total cohort). Median age not provided. No comparison group *Treatment type:* surgery*-*APR (100%). All women previously had RT-specific technique not disclosed	Sexual dysfunction, dyspareunia, vaginal symptoms and orgasm function (FSFI^c^) assessed at median 40 m (range 11–266) post-treatment completion	24% of women were no longer sexually active after APR for anal cancer. Of those who were sexually active after APR, 50% claimed to feel sexually aroused and reached orgasm without any issues Mean overall FSFI scores for women who were sexually active before surgery were 15.6, below the recommended cut-off of 26.55 indicating sexual dysfunction
Das (2010), USA	To evaluate long-term QOL in patients treated with RT or chemo-RT for SCC of the anal canal	*Design:* Retrospective cross-sectional study *Sample:* 26 women from a total cohort of 32 patients. Median age 51. No comparison group *Treatment type:* Concurrent chemo/RT (97%) and RT alone (3%). 3D-CRT and likely non-conformal techniques used	Orgasm function (FACT-C^d^; MOS-SPS^e^) assessed at median 5y (range 3–13) post-treatment completion	70% of female respondents reported difficulty in having an orgasm Overall median score of 61/100 was recorded for the MOS-SPS, where higher scores indicate worse function
DeFrancesco (2016), UK	To evaluate the potential impact of concurrent chemotherapy and IMRT on long-term consequences in patients diagnosed with anal cancer	*Design:* Prospective cross-sectional study *Sample:* 21 women from a total cohort of 27 patients. Median age 58 (47–84). No comparison group *Treatment type:* concurrent chemo/IMRT (100%)	Dyspareunia and vaginal symptoms (Pelvic Symptom Questionnaire) assessed at 1- to 3.5-y post-treatment completion	33% of women reported vaginal dryness. 33% of women claimed that treatment for anal cancer did not affect their sexual relationships. 57% declined to answer this question or stated ‘not applicable’
Fakhrian (2013), Germany	To report chronic adverse events according to the CTCAE^f^ after chemoradiotherapy and analyse their correlation with QOL	*Design:* Retrospective cross-sectional study *Sample:* 32 women from total cohort of 42. Median age 64 (50–86). No comparison group *Treatment type:* concurrent chemo/RT (89%), RT alone (7%), and chemo alone (4%). Techniques not disclosed	Dyspareunia and vaginal symptoms (FACT-C^d^; CTCAE^f^) assessed at median 68 m (range 9–222) post-treatment completion	About 20% were embarrassed to discuss sexual symptoms with their clinicians. Over 70% were later found to have some degree of dyspareunia
Ginesi (2023), USA	To evaluate QOL in patients treated with chemoradiotherapy for anal cancer using a disease-specific measure	*Design:* Retrospective cross-sectional *Sample:* 18 women from total cohort of 21 patients. No comparison group *Treatment type:* Radiotherapy techniques not disclosed	Sexual function (EORTC QLQ-ANL27^g^) was assessed at unknown time points post-treatment completion	The median sexual function score for women was significantly lower (worse) than male participants
Jephcott (2004), Canada	To assess QOL in patients at a single institution who have undergone non-surgical treatment for anal cancer	*Design:* Retrospective cross-sectional study *Sample:* 37 women from total cohort of 50 patients. Median age 68.5 (45–89). General QOL scores compared with 50 healthy volunteers recruited locally to the recruitment site that had not undergone any treatment to their abdomen or pelvis *Treatment type:* Concurrent chemo/RT (100%). RT delivered in two phases with a gap between phases. 3D-CRT and likely non-conformal techniques used	Sexual dysfunction (EORTC QLQ-CR38^h^) assessed at median 62 m (range 28–146) post treatment completion	When compared to age- and sex-matched controls, women with anal cancer were found to have mean score difference of 30 points, indicating worse sexual function. Response rates were similar between the two groups (43%:41%)
Knowles (2015), Scotland	To evaluate the prevalence of long-term urinary, bowel, sexual dysfunction, and overall QOL in patients treated with combined chemotherapy and radiotherapy for anal cancer	*Design:* Retrospective cross-sectional study *Sample:* 31 women from total cohort of 42 patients. Median age 54 (SD 10.8). No comparison group *Treatment type:* Concurrent chemo/RT (98%) and RT alone (2%). 3D-CRT used	Dyspareunia, vaginal symptoms and sexual problems (EORTC QLQ-CR38^h^) assessed at median 63.8 m (41.4–93.5) post-treatment completion	7/31 female participants answered questions about sexual function. 85% reported vaginal dryness and 100% reported pain during intercourse
Koerber (2019), Germany	To evaluate the occurrence of acute and chronic side effects including QOL and sexual behaviour before, during, and after treatment	*Design:* Retrospective cross-sectional study *Sample:* 47 women (total cohort). Median age 55 (40–86). No comparison group *Treatment type:* 3D-CRT (30%) and IMRT (70%)	Dyspareunia and vaginal symptoms (study-specific questionnaire) assessed at 3y (range 1–16) post-treatment completion	3D-CRT was associated with more frequent and severe symptoms compared to IMRT in all sexual function and intimacy domains
Provencher (2010), Canada	To evaluate QOL of patients with anal cancer treated with 3D-CRT and 2D (non-conformal) radiation techniques	*Design:* Retrospective cross-sectional study *Sample:* Cohort of 30 patients with no gender breakdown provided. Median age 53. Six patients (10%) were HIV positive, which likely contributed to the young median age. No comparison group *Treatment type:* 100% treated with concurrent chemo/3D-CRT and likely non conformal techniques (based on study timeframe)	Dyspareunia and sexual function (EORTC QLQ-CR29^a^) assessed at median 3y (range 6 m- 10y) post-treatment completion	65% of women had no interest in sex following treatment for anal cancer. Of those who showed interest, 50% experienced dyspareunia during intercourse. Number of female respondents not outlined
Rooney (2024), USA	To create and compare multiple dosimetric-based models to identify predictors of patient-reported sexual dysfunction and to determine an optimal dosimetric constraint that could be used as a reasonable clinical goal to reduce the risk of patient-reported dyspareunia	*Design:* Retrospective cross-sectional study *Sample:* 90 women (total cohort). Median age 64. No comparison group *Treatment type:* Median 54 Gy of radiation delivered using IMRT over median 27 fractions	Dyspareunia and sexual function (FSFI^c^) assessed at median 58 m post-treatment completion	Median overall FSFI score was 20.1 (out of a possible 36) which is below the recommended threshold of 26.55, indicating sexual dysfunction Median FSFI sub-score for pain was 2.4 (out of a possible 6)
Sauter (2022), Germany	To assess self-reported long-term QOL after combined radiation and chemotherapy, and to identify patient-, disease-, and therapy-related factors associated with QOL	*Design:* Retrospective cross-sectional study *Sample:* 38 women from total cohort of 52 patients. Median age 64.5 (range 48–87). A reference group from the general population was included in this study. Scores were age and sex matched for comparison *Treatment type:* concurrent chemo/RT (71%) and RT alone (29%). 3D-CRT and IMRT techniques used- outcome data presented separately for each treatment type	Sexual function (EORTC QLQ-ANL27^g^) assessed at median 71 m (range 7–176 m) post-treatment completion	Women who received a higher median dose of radiation (> 55.8 Gy) recorded statistically significantly higher sexual function mean scores compared to women who received < 55.8 Gy (p = 0.000)
Sunesen (2015), Denmark	To conduct a multicentre cross-sectional questionnaire study to assess the occurrence of symptoms and distress related to anorectal, urinary, and sexual dysfunction among colostomy-free survivors after curative intent radiotherapy or chemoradiotherapy for anal cancer	*Design:* Retrospective cross-sectional study *Sample:* 76 women from total cohort of 84 patients. Median age 58 (range 35–85). No comparison group *Treatment type:* 3D-CRT (100%) with concurrent chemo (29%)	Dyspareunia, sexual function and vaginal symptoms (study-specific questionnaire) assessed at median 33 m (range 5–92 m) post-treatment completion	Over 50% reported great distress from vaginal dryness and pain/discomfort during intercourse 61% reported ‘very decreased’ or no sexual desire 76% of reported overall dissatisfaction with their sex life
Welzel (2011), Germany Data from 1990 to 2006	To assess self-reported long-term QOL experienced by anal cancer patients after chemoradiotherapy and to identify patient- and disease-related factors associated with QOL	*Design:* Retrospective cross-sectional study *Sample:* 37 women from total cohort of 52 patients. Median age 62 (35–87). General QOL scores were compared with scores previously published from a German reference group *Treatment type:* 3D-CRT (100%)	Sexual dysfunction (EORTC QLQ-CR38^h^) assessed at median 36 m (range 5–137 m) post-treatment completion	27% of female participants (*n* = 10/37) responded to the question about sexual dysfunction. A radiotherapy dose > 50.4 Gy was associated with worse female sexual function
Yerramilli (2020), USA	To evaluate patient-reported sexual function, QOL, and mood among patients with anal SCC who received modern dose-painted IMRT	*Design:* Prospective cross-sectional study *Sample:* 34 women from total cohort of 50 patients. Median age 65 (44–86). No comparison group *Treatment type:* Concurrent chemo/IMRT (100%)	Dyspareunia, sexual function and vaginal symptoms (EORTC QLQ-CR29^a^; FSFI^c^) assessed at median 36 m post-treatment completion	85% of women who responded to FSFI questionnaire recorded scores consistent with sexual dysfunction. Median sub-scale scores for lubrication were (1.4/6) and vaginal pain (1.6/6) where lower scores indicate worse function Lowest response rates to questions about sexual satisfaction (62%) and dyspareunia (65%)

Abbreviations: *HRQL* health-related quality of life, *w* week, *m* month, *y* year, *IMRT* intensity-modulated radiation therapy, *CRT* chemoradiotherapy, *SCCA* squamous cell carcinoma of the anus, *RT* radiation therapy, *SCC* squamous cell carcinoma, *3D-CRT* 3-dimensional conformal radiation therapy, *QOL* quality of life, *Gy* Grey, *ASCC* anal squamous cell carcinoma

^a^European Organisation for Research and Treatment of Cancer Quality of Life Questionnaire for use among patients with Colorectal Cancer-29 checklist items

^b^Patient Reported Outcomes Measurement Information System

^c^Female Sexual Function Index assesses sexual function in response to 19-items and six domains based on the previous 4 weeks’ activity, while dyspareunia is assessed by three single questions which constitute one domain, labelled pain

^d^Functional Assessment of Cancer Therapy- Colorectal —asks respondents to rate their level of sexual satisfaction, regardless of their current level of sexual activity, based on the previous 7 days

^e^Medical Outcomes Study Sexual Problems Scale

^f^Common Terminology Criteria for Adverse Events

^g^European Organisation for Research and Treatment of Cancer Quality of Life Questionnaire for use among patients with Anal Cancer-27 checklist items

^h^European Organisation for Research and Treatment of Cancer Quality of Life Questionnaire for use among patients with Colorectal Cancer- 38 checklist items. According to EORTC, sexual function is the extent to which a woman was interested in sex over the last 4 weeks; dyspareunia refers to pain or discomfort during intercourse over the last 4 weeks [17]

**Table 2:** Summary of findings from cohort studies (*n* = 9)

First author (year) and country	Study aim	Design, sample size, comparison group, treatment type	PROs assessed (PROM used), time points	Key findings
Axelsson (2024), Sweden	To assess the occurrence of long-term urinary and sexual dysfunction at 3 and 6 years after diagnosis	*Design:* Prospective cohort study *Sample:* 281 women from a total cohort of 388 participants. 159 responded at 3- and/or 6-year time point while 122 were deceased or declined participation. No comparison group *Treatment type:* Radiation techniques not disclosed. 88% of total sample (including men) received chemotherapy and radiotherapy in some combination	Sexual dysfunction (study-specific 260-item questionnaire) assessed at 3-year and 6-year post-diagnosis	There was a 2% increase (30 to 32%) in the number of women able to respond to questions about dyspareunia (suggesting sexual activity) 68% of women denied having felt sexually aroused at 3- and 6-year post-treatment completion There was an 8% increase (from 62 to 70%) in the number of women who did not know about the elasticity of their vagina between 3- and 6-year post-treatment completion (suggesting sexual inactivity) 78% of women responded at 3- and/or 6-year timepoints 67% of women were deceased or declined to participate Descriptive analysis only
Gilbert (2020), UK	To evaluate the impact of IMRT on patient-reported toxicity, including HRQL at 1 year in a national anal cohort supplemented with oncological outcomes	*Design:* Prospective national cohort study *Sample:* 135 women from total cohort of 187 patients. Median age 61 (29–90). No comparison group *Treatment type:* concurrent chemo/IMRT (96%) and RT alone (4%)	Sexual function and dyspareunia (EORTC QLQ-CR29^a^) assessed at baseline and 1y post-treatment completion	Sexual function improved by 3.3 mean score points between time points Dyspareunia worsened by 10.2 mean score points between time points PRO completion for whole sample (men and women combined) declined from 61.5 to 30.5% between time points No findings were of statistical significance
Han (2014), Canada	To evaluate the acute and long-term toxicities, QOL, and disease outcome in patients treated with primary IMRT and concurrent chemotherapy	*Design:* Prospective cohort study *Sample:* 30 women from total cohort of 58 patients. Median age 56 (39–88). No comparison group *Treatment type:* concurrent chemo/IMRT (96.5%) and RT alone (3.5%)	Sexual function and dyspareunia (EORTC QLQ-CR29^a^) assessed at baseline, end of treatment, 3-month, 6-month, and 12-month post-treatment completion	Mean sexual function scores improved from baseline (77) to end of treatment (92), deteriorated at 3-month post-treatment completion (77), improved at 6-month post-treatment completion (83) and 12-month post-treatment completion (84) Mean scores indicate the same sexual function at 3-month post-treatment completion compared to baseline Mean dyspareunia scores worsened from baseline (19) to end of treatment (44), showed improvements at 3-month (36) and 6-month (27) post-treatment completion and then deteriorated by 12-month post-treatment completion (42). Worst dyspareunia scores were recorded at end of treatment (44) and 12-month post-treatment completion (42) Response rates for the whole sample (men and women combined) dropped from 94% at baseline to 67% 1-year post-treatment completion No findings were of statistical significance
Joseph (2016; 2023), Canada	To prospectively evaluate the impact of IMRT on QOL during and post-treatment	*Design:* Prospective cohort study *Sample:* 36 women from total cohort of 54 patients. Median age 57 (37–83). No comparison group *Treatment type:* Concurrent chemo/helical tomotherapy (IMRT) (100%)	Sexual function and dyspareunia (EORTC QLQ-CR29^a^) assessed at baseline, end of treatment, 6w, 12w, 6 m, 12 m, 2y, 3y, 4y, and 5y post-treatment completion Data is reported inconsistently across two papers. Authors were contacted for clarification but did not respond	Sexual function and dyspareunia findings between baseline and 12 m post-treatment completion cannot be interpreted due to data discrepancies. Sexual function increased (improved) by 5y post-treatment completion compared to baseline. Dyspareunia worsened by 5y post-treatment completion compared to baseline
Kronborg (2018), Denmark	To collect coinciding clinical graded and patient-reported outcome data pre- and post-treatment and correlate agreement between datasets	*Design:* Prospective cohort study *Sample:* 73 women from total cohort of 100 patients. Median age 62.9 (SD 11). No comparison group *Treatment type:* Concurrent chemo/IMRT (59%) RT alone (41%)	Dyspareunia and vaginal symptoms (EORTC QLQ-CR29^a^) assessed at baseline, end of treatment, and 2- to 4-week post-treatment completion	Graphs suggest worsening dyspareunia at latter time points compared to baseline. Raw data is not provided Response rates for whole sample were 79–89% at end of treatment and 48% 2–4 weeks post-treatment completion
Lefevre (2023), Denmark	To report on patient-reported and clinician-graded outcomes in female patients with SCCA 1 year after treatment with IMRT	*Design:* Cohort study *Sample:* 201 women from total sample 248. Median age 64 (whole sample). No comparison group *Treatment type:* Concurrent chemo/IMRT (79%) and RT alone (21%)	Dyspareunia, sexual function, and vaginal symptoms (EORTC QLQ-CR29^a^) assessed at baseline and 1y post-treatment completion	Mean sexual function scores decreased (suggesting worse sexual function) from baseline (17.3) to 1-year post-treatment completion (16.5) Mean dyspareunia scores increased (suggesting worse dyspareunia) from baseline (13.8) to 1-year post-treatment completion (26.6) Response rates to questions about sexual function and dyspareunia dropped by 3% and 19% between baseline and 1-year post-treatment completion No findings were of statistical significance
Savoie (2023), USA	To describe sexual function and sexual activity among women who underwent definitive treatment for ASCC	*Design:* Cohort study with some cross-sectional findings also reported. No comparison group *Sample:* 21 women (total cohort). Median age 63. No comparison group *Treatment type:* 95% of women treated with concurrent chemo/RT. RT techniques not disclosed. 9% of women underwent surgical intervention—details of which are undisclosed	Data reported within 1 year, 1–2 years, and beyond 2 years Dyspareunia, sexual function and vaginal symptoms (EORTC QLQ-CR29^a^; EORTC QLQ-ANL27^g^; FSFI^c^) assessed at 1y, 1–2 years, and beyond 2 years	Median FSFI scores declined gradually from 21.8 (< 1 year) to 20.5 (1–2 years) to 17.0 (> 2 years) where lower scores indicate worse sexual function Median FSFI scores were worst at > 2-year post-treatment completion in women treated for anal cancer (17) compared to those treated for colon (23.5) and rectal (22) Response rates to FSFI were 29%, 14%, and 10% at specified time points
Tang (2015), USA	To evaluate QOL in patients treated with IMRT for anal cancer	*Design:* Prospective cohort study with findings from one time point included in analysis *Sample:* 27 women from total cohort of 34 patients. Median age 58 (range 49–80). No comparison group *Treatment type:* Concurrent chemo/IMRT (100%)	Orgasm function (FACT-C^c^; MOS-SPS^e^) assessed at 3y (range 1–16) post-treatment completion	50% of women had trouble reaching an orgasm between 49- and 80-month post completion of treatment 59% response rate to this question
Taylor (2022), USA	To evaluate long-term changes to bowel, urinary, and sexual function in patients treated with combined modality therapy for anal SCC	*Design:* Cohort study *Sample:* 104 women from total cohort of 143 patients. Median age (total cohort) 62. No comparison group *Treatment type:* 100% combined modality therapy with IMRT	Sexual arousal and lubrication (study-specific questionnaire) assessed at 3 m, 6 m, 12 m, 18 m, 24 m, 3y, 4y, and 5y post-treatment completion	Graphs suggest improved sexual arousal over time, but raw data is not provided Response rates for whole sample were: 13%; 25%; 29%; 13%; 26%; 20%; 13%; 2.8% for each time point listed

**Table 3:** Sexual function and intimacy outcomes reported by study

Study first author (year of publication)	Sexual interest/function/desire/arousal	Dyspareunia and pain/discomfort during sex	Sexual dysfunction	Inability to achieve an orgasm	Vaginal pain	Vaginal dryness	Vaginal or vulval itching and burning	Narrowing and shortening of the vagina
Axelsson (2024)	•	•	•	•		•		
Bentzen (2013)	•	•						
Corrigan (2022)	•			•	•	•		
Corte (2011)	•	•		•	•	•		
Das (2010)				•	•			
DeFrancesco (2016)					•	•		
Fakhrian (2013)		•			•		•	
Gilbert (2020)	•	•						
Ginesi (2023)	•							
Han (2014)	•	•						
Jephcott (2004)			•					
Joseph (2016; 2023)		•						
Knowles (2015)			•					
Koerber (2019)		•			•	•		
Kronborg (2018)		•			•	•		
Lefevre (2023)	•	•						
Provencher (2010)	•	•						
Rooney (2024)	•	•						
Sauter (2022)	•	•						
Savoie (2023)	•	•						
Sunesen (2015)	•	•				•		•
Tang (2015)				•				
Taylor (2022)	•							
Welzel (2011)			•					
Yerrmailli (2020)	•	•		•		•		

### Study selection and eligibility criteria

Studies published in English were included if:

- They were prospective, retrospective, cross-sectional, or qualitative primary research.
- Study participants were cis-gendered females diagnosed with and treated/currently receiving treatment for anal cancer. Treatment was not restricted to concurrent chemoradiotherapy and could include surgical procedures.
- Treatment of participants included the use of conventional and conformal radiation techniques
- Sexual function and/or intimacy was assessed using Patient-Reported Outcome Measures (PROMs).
- Approved by a relevant ethics board.

We excluded studies if sex and intimacy outcome data for women with anal cancer was unable to be separated from total sample findings (e.g. where data was reported alongside male participants and/or other tumour groups such as colorectal or rectal cancer) or if conventional radiotherapy was the only radiation technique used.

Two reviewers independently reviewed titles and abstracts for eligibility, obtained full texts, and conducted independent assessments. The reviewers discussed discrepancies until consensus was reached. They entered full texts into a shared EndNote library to aid data extraction and analysis.

### Data extraction and analysis

Study aims, design, sample demographic and clinical characteristics, outcomes assessed, PROMs used, and results were extracted from included studies on a data extraction template specifically designed for this review. In longitudinal studies, reviewers extracted relevant data for all assessment time-points. Where data were missing, reviewers contacted corresponding authors for clarification.

Terminology was used interchangeably between study authors and within study PROMs to describe the same or similar Patient-Reported Outcomes (PROs) related to sexual function and intimacy. For example, ‘sexual function/interest’ was shortened to ‘sexual function’ by different authors using the same PROM. To aid data analysis, we grouped similar sexual function and intimacy PROs during data extraction. A single reviewer conducted data extraction which was cross-checked by a second reviewer. Reviewers discussed discrepancies until consensus was reached.

Following consultation with a university biostatistician, we used a narrative synthesis approach to analyse the findings [18] as a meta-analysis of effect estimates was not considered feasible due to small study sample sizes, low response rates, and high rates of participants lost to follow-up. We synthesised relevant sexual function and intimacy PROs by treatment type (3D-CRT or IMRT) and time between PRO assessment and completion of treatment.

### Quality appraisal

Two reviewers independently assessed study quality using cross-sectional and cohort study checklists from the Joanna Briggs Institute (JBI) [19]. The checklists contain eight and eleven items respectively. Reviewers categorised studies yes/no/unclear for each item. Studies that satisfied all checklist items indicated a low risk of bias based on their study design. Studies that failed to satisfy one or more checklist items or where the data were regarded as unclear were deemed at risk of containing bias.

## Results

### Summary of included studies

**Fig. 1:**
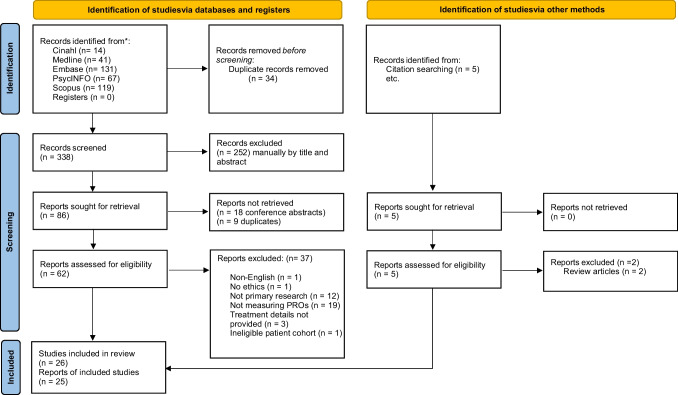
PRISMA flow chart [15]^a^

From 338 abstracts retrieved, we included 26 articles reporting patient-reported sexual function and intimacy outcomes for 25 studies; two articles reported on the same study at different time-points [20, 21]. Studies were cross-sectional (*n* = 16) or prospective cohorts (*n* = 10; Fig. 1). Studies included 1546 women from ten countries (Tables 7 and 9). Sample sizes, where provided, ranged from 21 to 281 female participants. Sample characteristics varied; four studies included female participants only, while 21 studies included men and women. Participants were recruited between 1-month and 19-year post-treatment completion. Nineteen different sexual function and intimacy PROs were assessed using ten different PROMs. Male and female were the only recognised genders across studies. Sexual orientation was not described.

### PROMs used to assess sexual function and intimacy outcomes

Table 3 and Supplementary Table 1 summarise the sexual function and intimacy PROs assessed and the PROMs used to assess them. The most commonly used PROMs were the European Organisation for Research and Treatment of Cancer (EORTC) suite of measures [17], the Functional Assessment of Cancer Therapy- Colorectal (FACT-C), and the Female Sexual Function Index (FSFI) [22]. These PROMs have been widely validated and are considered suitable for use within cancer research. Three studies developed study-specific questionnaires [23–25].

### Primary cancer treatment

One study included APR in the context of recurrent disease [26], while the rest treated participants with radiotherapy and chemotherapy. Radiotherapy techniques used within studies varied between conventional and conformal techniques. Patient-reported sexual function and intimacy findings (summarised in Tables 7 and 9) are distinguished by radiation technique delivered within each study. It is important to highlight that at least eight studies use treatment modalities that are now outdated.

Details of radiotherapy techniques was absent in four studies [27–30]. Two studies compared sexual function and intimacy PROs for both IMRT and 3D-CRT techniques [24, 31]. Thirteen studies reported sexual function and intimacy PROs for participants treated with IMRT [20, 21, 23, 32–41], three studies for participants treated with 3D-CRT [25, 42, 43] and three studies pooled participant findings for those treated with 3D-CRT and older (non-conformal) techniques [44–46].

### Effect of IMRT on female sexuality and intimacy

#### Dyspareunia and sexual function

Dyspareunia and sexual function were the two most frequently assessed sexual function and intimacy PROs across included studies. Table 2 summarises findings from four longitudinal studies (*n* = 30–201), where consistently higher rates of dyspareunia were reported over time using the EORTC QLQ-CR29 [20, 21, 34, 38, 39]. Kronborg and colleagues [37] found higher rates of dyspareunia at end of treatment and 2–4 weeks post-treatment completion using the same assessment tool. They reported findings graphically but did not provide results of any statistical analysis. Two studies reported interim improvements in dyspareunia at 3-month, 6-month, and 4-year post-treatment completion [20, 21, 38]. No dyspareunia findings were statistically significant. High non-response rates were observed (Table 2).

Two longitudinal studies (*n* = 30–135) found improvements in sexual function over time using the EORTC QLQ-CR29 (Table 2) [38, 39]. Savoie and colleagues [35](*n* = 6) reported a consistent decline in sexual function from baseline to 1- and 2-year post-treatment completion. Taylor et al. [23] (*n* = 104) reported improved sexual arousal from baseline to 5-year post-treatment completion using a study-specific questionnaire, although response rates of 2.8% at 5-year post-treatment completion must be considered. None of these findings was statistically significant.

Three cross-sectional studies (*n* = 34–90) reported mean scores consistent with dyspareunia/vaginal pain at median times of 36-, 51-, and 58-month post-treatment completion using the FSFI [32, 33, 41]. Findings from DeFrancesco and colleagues’ study of 21 women [40] showed 24% reported pain affected or stopped sexual activity while 57% reported that this question was not applicable to them using the Pelvic Symptom Questionnaire. Cross-sectional findings from Savoie’s cohort study (*n* = 17) [35] showed 42% of women reported pain during intercourse at median 14-month post-treatment completion.

Three cross-sectional studies (*n* = 34–90) found sexual function below the validated threshold of 26.55 on the FSFI [22] at median 36- [32], 51- [33], and 58 month [41] post-treatment completion. Savoie and colleagues [35] found 35% of women reported no interest in sex at median 14-month post-treatment completion.

#### Orgasm

Three studies reported orgasm outcome. Tang and colleagues (*n* = 27) found 50% of participants reported a decline in orgasm function at median 33-month post-treatment completion using the MOS-SPS [36]. Corrigan et al. and Yerramilli et al. reported mean scores indicative of orgasm function at 36-month [32] and 51-month [33] post-treatment completion using the FSFI. Respective sample sizes (90:34) and response rates (57%:91%) must be considered when interpreting these findings.

#### Vaginal dryness

Two cross-sectional studies (*n* = 34–90) reported low mean scores consistent with vaginal dryness at 36-month [32] and 51-month [33] post-treatment completion using the FSFI. Response rates (57%:88%) must be considered when interpreting these findings. DeFrancesco et al. found 33% (*n* = 7) reported vaginal dryness and 14% (*n* = 3) reported vaginal dryness that interfered with sexual intercourse between 1-and 3-year post-treatment completion using the Pelvic Symptom Questionnaire [40].

### Effect of non-IMRT and unknown/undisclosed radiation techniques on female sexuality and intimacy

#### Dyspareunia and sexual function

Longitudinal findings from Axelsson et al. (Table 2) highlight ongoing dyspareunia concerns for women at 3- and 6-year post-treatment completion [28]. Bentzen’s cross-sectional study (*n* = 101) reported mean scores consistent with dyspareunia at a median 66-month post-treatment completion using the EORTC QLQ-CR29 [29]. Two additional cross-sectional studies (*n* = 21–73) found 60% had severe dyspareunia that made intercourse impossible, and 92% had chronic dyspareunia at median 33- and 36-month post-treatment completion using study-specific questionnaires [24, 25]. Knowles et al. found 100% of women (*n* = 31) reported dyspareunia at a median 64-month post-treatment completion using EORTC QLQ-CR38 [43].

Bentzen et al. (*n* = 101) reported low mean scores indicating impaired sexual function at median 66-month post-treatment completion using the EORTC QLQ-CR29 [29]. These findings were statistically significant when compared to age- and sex-matched controls recruited from the general population. Provencher et al. (*n* = 32) found 65% reported no interest in sex at a median 3-year post-treatment completion using the same questionnaire but no comparison group [46]. Additionally, further cross-sectional findings (*n* = 76) showed 61% reported a ‘very decreased’ or ‘absent’ sex drive while 23% were overall satisfied with their sex life at median 33-month post-treatment completion, using a study-specific questionnaire [25]. Three cross-sectional studies (*n* = 31–37) reported mean scores consistent with sexual dysfunction at median 36- [42], 62- [44], and 64-month [43] post-treatment completion using the EORTC QLQ-CR38.

#### Orgasm

Das et al.’s cross-sectional study (*n* = 26) found 70% reported difficulties in having an orgasm as ‘somewhat’ or ‘very much’ a problem at median 5-year post-treatment completion using the Medical Outcomes Study Sexual Problems Scale (MOS-SPS) [45]. Longitudinal findings for patient-reported orgasm could not be synthesised due to inaccurate reporting [28].

#### Vaginal dryness and associated vaginal health issues

Longitudinal findings (*n* = 281) collected by a study specific questionnaire indicated that high rates of women are not sexually active at 3- and 6-year post-treatment completion [28]. A cross-sectional study (*n* = 76) also using a study-specific questionnaire found 59% reported vaginal dryness and 73% reported a narrower and shorter vagina at median 33-month post-treatment completion [25]. Fakhrian and colleagues (*n* = 32) also found that 22% reported vaginal dryness, itching, and burning at median 68-month post-treatment completion using the FACT-C and the Common Terminology Criteria for Adverse Events [27].

### Effects of APR on female sexuality and intimacy

Corte et al. (*n* = 21) reported sexual function PROs for respondents who were sexually active prior to APR at a median 40-month post-treatment completion [26]. 76% were sexually active after surgery, 62% were unable to have an orgasm, 71% reported vaginal dryness, 57% reported dyspareunia, and 43% reported discomfort during intercourse [22].

### Comparing radiation techniques

Two cross-sectional studies (*n* = 47;38) compared sexual function and intimacy PROs for participants treated with 3D-CRT and IMRT using a study-specific questionnaire [24] and EORTC QLQ-ANL27 [31]. Sauter et al. found statistically significantly higher sexual function scores in the IMRT-treated group compared to the 3D-CRT-treated group at median 71-month post-treatment completion [31]. Koerber et al. found patients treated with 3D-CRT reported 7–20% more cases of vulval pruritus, ulcerations, stenosis, and colpitis during treatment compared to those treated with IMRT at median 3-year post-treatment completion [24]. This study also reported a statistically significant difference in severe, chronic vaginal dryness, with 83% of 3D-CRT-treated patients reporting this outcome compared to 42% IMRT-treated patients.

### Quality appraisal/risk of bias

**Fig. 2:**
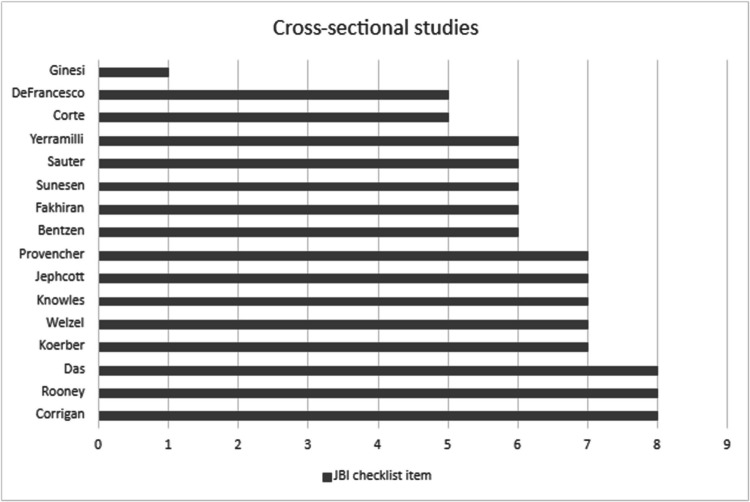
Quality appraisal of cross-sectional studies^b^ using JBI Checklists [19]. Total scores illustrated. ^b^Maximum possible score for cross-sectional studies is 8

**Fig. 3:**
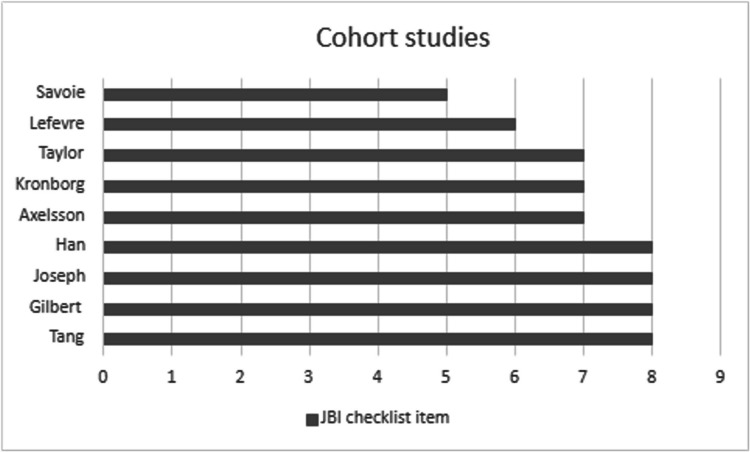
Quality appraisal of cohort studies^c^ using JBI Checklists [19]. Total scores illustrated. ^c^Maximum possible score for cohort studies is 11

Figures 2 and 3 illustrate the quality of each study. Three cross-sectional studies satisfied all quality appraisal items, indicating a low risk of bias [33, 41, 45]. One study failed to meet seven of the eight quality items [30]. No cohort study met quality item 9 (n lost to follow-up) or 10 (strategies utilized to address missing data). All longitudinal studies incompletely reported follow-up rates, had high drop-out rates, and failed to describe any actions taken to mitigate loss to follow-up [20, 23, 28, 34–39]. No cohort study included a comparison group; therefore, quality items 1 and 2 were unachievable for these studies.

### PROM response rates

Table 2 reports response rate declines over time (19–70%) for longitudinal studies. Sixty to eighty percent of missing data from cross-sectional studies were mostly observed on questions about sex and intimacy [24, 42, 43] (Table 1). Non-response rates to questions about dyspareunia were between 57 and 70% [27, 40]. One study reported a 100% completion rate for items about sex and intimacy [46].

## Discussion

This review synthesised the available evidence on patient-reported impacts of anal cancer treatment on female sexual function and intimacy. Findings show that overall sexual function was negatively impacted, resulting in dyspareunia, impaired sexual function, and a sequelae of side-effects involving the vagina, including reduced lubrication and elasticity. When compared to age- and sex-matched controls, women with anal cancer reported statistically significantly higher rates of dyspareunia and impaired sexual function, [29] with impacts persisting for up to 5-year post-treatment completion [33, 41]. The overwhelming majority of female participants in the Swedish ANCA study (*n* = 159) reported that intercourse was no longer part of their sex life at 3- and 6-year post-treatment completion, highlighting the possible detrimental long-term effects on sexual wellbeing [28].

Where 3D-CRT and IMRT radiation treatment protocols were directly compared, the outdated 3D-CRT modality was associated with statistically significantly worse sexual function, physical vaginal health, and overall sexual satisfaction [24, 31]. Outcomes assessed in the studies in this review such as dyspareunia, sexual function, and physical changes to the vagina are a narrow representation of the possible impacts of treatment on sexual function and intimacy. Outcomes were largely influenced by the chosen study questionnaires, most of which are designed to assess the impact of treatment on HRQL rather than on sexual function, specifically. It is also critical to acknowledge the side-effects that are not analyzed as part of this review such as faecal incontinence, flatulence, and skin changes, and realise the potential and varying impact that these side-effects will have on sexual function and intimacy.

Despite extensive discussion in clinical settings and conference forums around the unmet supportive care needs of anal cancer survivors, the literature is sparse and datasets are largely incomplete, suggesting that sex and intimacy outcomes are not prioritised in anal cancer research [47]. Validated PROMs designed specifically for use among people with anal cancer have only been available since 2023 and were never used alongside 3D CRT treatment modalities [17]. Several large studies were excluded from this review as findings were pooled with men or other tumour groups making it impossible to extract findings specific to women with anal cancer [13, 14]. In the few studies that specifically aimed to assess sexual function and intimacy outcomes, findings were limited by small sample sizes (*n* =  < 50), high risk of biases, and inconsistent reporting. Predominantly, qualitative lived experience evidence concerning women with anal cancer is derived from quantitative studies that collected open-ended responses within surveys [11]. These studies tend to include men and women with various cancers such as colorectal, penile, and vulva cancer [48, 49]. Personal accounts and conference abstracts have attempted to describe associated psychosocial concerns including stigma, family relationships, and sexuality [50, 51] but lacked rigour, limiting generalisability of findings. Such gaps limit understanding and ability to adequately prepare women with anal cancer to make informed decisions about treatment and supportive care pathways.

These review findings must be interpreted within the context of the evolution of anal cancer treatment over the past four decades where gold standard treatment now includes radio sensitising adjuvant chemotherapy which has contributed to improved outcomes and reduced toxicities. At least five studies reported PROs for women treated with 3D-CRT and many women currently living with and accessing specialist services received treatment using this modality. Therefore, it remains important for clinicians to understand their needs profile to ensure appropriate care and support. Ongoing developments in radiation techniques used during planning, simulation, and treatment have been shown to reduce toxicity to surrounding organs at risk, with further advancements involving sparing of the bulboclitoris (clitoris and vestibular bulbs) on the horizon [52, 53]. Given the persistent rise in anal cancer diagnoses among women [3], such consideration of female erectile tissues during radiation planning is a novel and welcomed approach where, traditionally, the vagina has been the focal point of sexual function.

The evolving landscape of HPV must also be considered, with increased rates of high-risk HPV and HPV-related cancers among transgender people [54]. Earlier diagnoses using HRA and artificial intelligence and surgical excisions coupled with ablative treatment of High Grade Squamous Intraepithelial Lesions (HSILs) are some examples of radiation-sparing approaches that should be considered in priority populations. Conversely, few advancements have been made in the way of supportive care. Correct and consistent use of vaginal dilators have been shown to reduce the impact of radiation-induced vaginal stenosis [55, 56] but data is scarce in the anal cancer setting and thus, there are no evidence-based guidelines available to support this practice.

### Limitations

There are methodological limitations pertaining to individual studies included in this review that should be considered when interpreting the findings. Most included studies had small sample sizes (e.g. *n* =  < 50), poor- and non-engagement from study participants, and fundamentally flawed study designs. Many studies were underpowered to detect statistically significant differences, leading to reliance on differences between mean scores, where available, to interpret any observed differences between groups or changes over time. Two longitudinal studies reported inconsistent or incomplete results between text and tables resulting in partial exclusion from the synthesis [20, 28]. Where studies used HRQL questionnaires without the primary aim of collecting sexual function and intimacy outcomes, feelings of embarrassment and shame prevented women from discussing intimate topics they perceived as uncomfortable or irrelevant to them, which contributed to participant disengagement [27, 40]. Previous review articles have similarly identified incomplete and inconsistent sexuality and intimacy data as significant barriers to synthesising the evidence, compounded by flawed study design, reporting methods and suspected biases [57, 58].

Recall bias was problematic in cross-sectional studies where participants were up to 19-year post-treatment completion [27]. Selection bias was influenced by small sample sizes, high non-response rates, and poor reporting of basic demographics, all increasing the likelihood that study cohorts may not be representative of the larger population of women with anal cancer. Studies where race/ethnicity was reported showed low participation from cultural and linguistically diverse (CALD) communities. Gender diversity or sexual orientation were not considered or described in studies, despite their relevance when discussing sex and intimacy.

Several limitations were also identified in how outcomes were assessed and reported in the studies included in this review. Heterogeneity in PROMs used to assess sexual function and intimacy outcomes made synthesis of findings across studies challenging. Three studies [30, 31, 35] used the recently validated anal cancer specific HRQL measure [17]. Just eight studies specifically aimed to collect sexual and/or intimacy outcomes while all other sex and intimacy-related data were reported through questions included in the HRQL questionnaires used [23, 26, 32, 33, 35, 36, 40, 45]. Further, it cannot be assumed that women understood what was being asked of them in every PROM. Respondents were asked to self-report outcomes including colpitis (inflammation of the vaginal mucosa), vaginal elasticity, and dyspareunia, without any background or contextual information about baseline sexual function, vaginal health, or sexual orientation. Where there were male and female participants, findings were limited by PROMs used and reporting methods. Pooling data across genders limits our understanding of the severity and extent of adverse outcomes for both men and women, limiting the evidence to inform the development of strategies and interventions to address these outcomes. There is a pressing need for a more consistent and accurate approach to identifying the frequency and severity of any ongoing problems affecting sexual function and intimacy. Comprehensive and timely assessment of treatment-related issues not only enables the healthcare team to intervene early but also helps the patient to realise the value and importance of these aspects of quality of life.

### Implications for future practice and recommendations for future research

Although it is well documented that sexual function is a common side-effect of oncological disease, many clinicians avoid this discussion for several reasons: lack of knowledge around treatment pathways and feelings of embarrassment, relying on other members of the multidisciplinary team to address such concerns [59]. This puts patients at increased risk of being under-informed about the impacts of their cancer treatment. Established guidelines such as the PLISSIT and extended PLISSIT models [60] provide a structured framework and stepwise approach that supports healthcare providers to engage in a comprehensive and non-confrontational discussion about sexual health and function. Clinician training and education to promote the uptake of these models at dedicated time-points throughout the treatment trajectory is imperative and must be factored into clinical training modules and University curricula going forward [61].

It is critically important that we provide evidence-based education and supports for women experiencing late-onset and permanent toxicities from pelvic chemoradiotherapy [11]. It is equally important to ensure that interventions are delivered using the most effective methods available. There is plausible evidence from the US to suggest that combined psychoeducation and psychotherapeutic approaches about the impact of cancer and treatment on sexual health and symptoms, and multidisciplinary sexual medicine programs may be successful ways to address broad-ranging sexual function concerns in people diagnosed with cancer [62]. In countries such as Australia which have vast and challenging landscapes with low-density populations outside of Metropolitan areas, Telehealth models should be considered to maximise healthcare accessibility and efficiency, while social media and interactive web-based platforms can build on these models to provide formal and informal psychological support and promote information sharing.

Tailored, gender-specific resources are a positive step towards de-stigmatising anal cancer and empowering anal cancer survivors to confidently engage in conversations about sex and intimacy during and after cancer. Timely intervention is critical and should be ongoing throughout the treatment trajectory and into recovery. Adopting a proactive approach may reduce the severity of consequences experienced by women and increase awareness of and access to supportive care services such as pelvic floor physiotherapy. Existing information resources around vaginal dilators, psychosexual therapy, and partner counselling may be transferrable across tumour streams in the oncology setting with adequate support and guidance [63]. Person-centred services dedicated to addressing the psychosexual impacts of cancer such as late-effects clinics and online-based services have been well received across the UK and Canada [64, 65].

Intimacy and sexual function remains one of the most frequently reported unmet needs for cancer survivors [47]. In order to gain insight into the severity and extent of these issues, qualitative or mixed methods should be explored and recruitment diversified to include minority groups such as CALD and sexually diverse populations. Collecting detailed demographic data about gender and sexual orientation is highly relevant in this area of research as healthcare professionals seek to understand how treatment impacts specific sexual practices in a variety of ways. To enhance and expand upon existing findings, future cohort studies should prioritise rigorous follow up protocols spanning beyond the customary 5- or 6-year timeframes that will promote understanding of the onset and severity of long-term, and potentially permanent, side effects.

Despite methodological limitations of the studies included, there is enough data to indicate that treatment for anal cancer negatively impacts overall sexual function, leading to dyspareunia, impaired orgasm, and reduced sexual and intimacy desire. Radiation techniques and time since completion of treatment play a role in determining the incidence and severity of sexual function-related outcomes assessed. Future research should prioritise qualitative studies and large comparative studies to explore the severity and extent of sexual changes and to better understand sexual function and intimacy trajectories.

## Supplementary Information

Below is the link to the electronic supplementary material.

ESM 1 DOCX (14.8 KB)

## Data Availability

No datasets were generated or analysed during the current study.

## Appendix

**Table 4:** Search terms used

Anus neoplasms Anal cancer Anus cancer Anal SCC Anal squamous cell carcinoma Anal carcinoma Anus tumor Anus carcinoma	Cancer treatment Antineoplastic agents Drug therapy Antineoplastic protocols Chemoradiotherapy Chemotherapy Radiotherapy Cytotoxic Anti-cancer Cancer surgery	Intimacy Marital Satisfaction Marriage Quality of life Health and life quality Psychological well-being Relationship Sexuality Psychosexual behaviour	Sexual function Sexual dysfunction Sexual functioning Sexual Behavior Female sexual function Sexual intimacy Sexual intercourse Sexual well-being Sexual wellbeing Sexual health Sexual satisfaction Sexual experiences Sexual problems Sexuality Sex	Female Girls Women Woman

**Table 5:** Search strategy used in MedLine

Search term 1	AND	Search term 2	AND	Search term 3
cancer treatment.mp. or exp Antineoplastic Agents/or drug therapy/or antineoplastic protocols/or chemoradiotherapy/or chemotherapy, adjuvant/or radiotherapy/or cytotoxic.mp. or anti-cancer*.mp. or cancer surgery.mp	AND	anal cancer.mp. or exp Anus Neoplasms/or anal squamous cell carcinoma.mp. or anal SCC.mp. or anal carcinoma.mp	AND	sexual funct*.mp. or exp “Quality of Life”/or exp Sexual Dysfunction, Physiological/or sexual dysfunction.mp. or exp Sexual Behavior/or exp Sexual Dysfunctions, Psychological/or female sexual function.mp. or sexual intimacy.mp

**Table 6:** Search strategy used in CINAHL

Search term 1	AND	Search term 2	AND	Search term 3
(((MH “Anus Neoplasms + ”) OR “anal cancer”) OR ((anus cancer) OR (anal SCC) OR (anal squamous cell carcinoma) OR (anal carcinoma)))	AND	(((MH “Intimacy”)) OR ((MH “Marital Satisfaction”) OR (MH “Marriage/PF”)) OR ((MH “Quality of Life + ”) OR (MH “Health and Life Quality (Iowa NOC) + ”) OR (MH “Psychological Well-Being”)))	AND	((sexual well-being OR sexual wellbeing OR sexual health OR sexual satisfaction) OR (sexual experiences) OR (sexual intercourse) OR ((MH “Sexual Dysfunction, Female + ”) OR (MH “Sexual Behavior + ”)) OR (sexual function) OR (“sexual functioning or sexual dysfunction or sexuality or sexual well-being or sexual problems or sex”))

**Table 7:** Search strategy used in Embase

Search term 1	AND	Search term 2	AND	Search term 3
anal cancer.mp. or anus cancer/OR exp anus tumor/di [Diagnosis] OR anus carcinoma/co, di [Complication, Diagnosis]	AND	exp sexual behavior/or exp sexuality/or exp sexual function/or exp sexual intercourse/or exp sexual dysfunction/	AND	intimacy/or “quality of life”/OR relationship*.mp

**Table 8:** Search strategy used in PsycINFO

Search term 1	AND	Search term 2
anal cancer.mp. OR anus neoplasms.mp. OR anal squamous cell carcinoma.mp. OR anal carcinoma.mp. (#158)	AND	exp Sexuality/or exp Female Sexual Dysfunction/or exp Sexual Function Disturbances/or exp Psychosexual Behavior/or exp “Quality of Life”/or sexual dysfunction.mp

**Table 9:** Search strategy used in Scopus

Search term 1	AND	Search term 2
anal AND cancer OR an* AND neoplasm* OR anal AND squamous AND cell AND carcinoma)	AND	(quality AND of AND life)

**Table 10:** Quality appraisal of cross-sectional studies according to JBI checklists

Study first author (year of publication)	Design	Inclusion criteria defined	Subjects and setting described	Exposure measured	Criteria used	Confounding factors	Strategies	Outcomes measured	Appropriate statistical analysis	Total
Bentzen (2013)	Cross-sectional	Y	Y	Y	Y	Y	N	U	Y	6/8
Corrigan (2022)	Cross-sectional	Y	Y	Y	Y	Y	Y	Y	Y	8/8
Corte (2011)	Cross-sectional	U	Y	Y	Y	Y	N	N	Y	5/8
Das (2010)	Cross-sectional	Y	Y	Y	Y	Y	Y	Y	Y	8/8
DeFrancesco (2016)	Cross-sectional	U	Y	Y	Y	Y	N	U	Y	5/8
Fakhrian (2013)	Cross-sectional	U	Y	Y	Y	Y	Y	N	Y	6/8
Ginesi (2023)	Cross-sectional	N	N	N	Y	U	N	U	U	1/8
Jephcott (2004)	Cross-sectional	Y	Y	Y	Y	Y	N	Y	Y	7/8
Knowles (2015)	Cross-sectional	Y	Y	Y	Y	Y	N	Y	Y	7/8
Koerber (2019)	Cross-sectional	U	Y	Y	Y	Y	Y	Y	Y	7/8
Provencher (2010)	Cross-sectional	U	Y	Y	Y	Y	Y	Y	Y	7/8
Rooney (2024)	Cross-sectional	Y	Y	Y	Y	Y	Y	Y	Y	8/8
Sauter (2022)	Cross-sectional	U	Y	Y	Y	Y	Y	N	Y	6/8
Sunesen (2015)	Cross-sectional	Y	Y	Y	U	Y	N	Y	Y	6/8
Welzel (2011)	Cross-sectional	U	Y	Y	Y	Y	Y	Y	Y	7/8
Yerramilli (2020)	Cross-sectional	N	Y	Y	Y	Y	N	Y	Y	6/8

**Table 11:** Quality appraisal of cohort studies according to JBI checklists

Study first author (year of publication)	Design	Similar sample groups	Similar exposure measured	Exposure measured	Confounding factors	Strategies	Free of outcome at start of study	Outcomes measured	Follow-up time reported and sufficient	Follow-up complete	Strategies to address incomplete FU	Appropriate statistical analysis	Total
Axelsson (2024)	Cohort			Y	N	N	Y	Y	Y	N	N	Y	7/11
Gilbert (2020)	Cohort			Y	Y	N	Y	Y	Y	N	N	Y	8/11
Han (2014)	Cohort			Y	Y	N	Y	Y	Y	N	N	Y	8/11
Joseph (2016; 2023)	Cohort			Y	Y	N	Y	Y	Y	N	N	Y	8/11
Kronborg (2018)	Cohort			Y	Y	N	Y	N	Y	N	N	Y	7/11
Lefevre (2023)	Cohort			U	U	N	Y	Y	Y	N	N	Y	6/11
Savoie (2023)	Cohort			U	Y	Y	U	Y	Y	N	N	Y	5/11
Tang (2015)	Cohort			Y	Y	Y	U	Y	Y	N	N	Y	8/11
Taylor (2022)	Cohort			Y	Y	Y	U	U	Y	N	N	Y	7/11

*Y*, yes; *N*, no; *U*, unclear; shaded = not applicable
