# Supplementary material for: The impact of anal cancer treatment on female sexuality and intimacy: a systematic review
Source: Support Care Cancer. 2025 Aug 15;33(9):788. doi: 10.1007/s00520-025-09791-1 (PMC12354548; doi:10.1007/s00520-025-09791-1)
Supplement: Supplementary file 1 — DOCX (14.8 KB) [file 520_2025_9791_MOESM1_ESM.docx]

| Supplementary Table 1: PROMs used in included studies to measure sex and intimacy outcomes | | | | | | | | | |
| --- | --- | --- | --- | --- | --- | --- | --- | --- | --- |
| **Study first author (year of publication)** | **EORTC QLQ-CR29^a^** | **EORTC QLQ-CR38^b^** | **EORTC QLQ-ANL27^c^** | **Study-specific questionnaires** | **FACT-C^d^** | **FSFI^e^** | **MOS SPS^f^** | **Pelvic symptom questionnaire** | **PROMIS^g^** |
| Axelsson (2024) |  |  |  |  |  |  |  |  |  |
| Bentzen (2013) |  |  |  |  |  |  |  |  |  |
| Corrigan (2022) |  |  |  |  |  |  |  |  |  |
| Corte (2011) |  |  |  |  |  |  |  |  |  |
| Das (2010) |  |  |  |  |  |  |  |  |  |
| DeFrancesco (2016) |  |  |  |  |  |  |  |  |  |
| Fakhrian (2013) |  |  |  |  |  |  |  |  |  |
| Gilbert (2020) |  |  |  |  |  |  |  |  |  |
| Ginesi (2023) |  |  |  |  |  |  |  |  |  |
| Han (2014) |  |  |  |  |  |  |  |  |  |
| Jephcott (2004) |  |  |  |  |  |  |  |  |  |
| Joseph (2016; 2023) |  |  |  |  |  |  |  |  |  |
| Knowles (2015) |  |  |  |  |  |  |  |  |  |
| Koerber (2019) |  |  |  |  |  |  |  |  |  |
| Kronborg (2018) |  |  |  |  |  |  |  |  |  |
| Lefevre (2023) |  |  |  |  |  |  |  |  |  |
| Provencher (2010) |  |  |  |  |  |  |  |  |  |
| Rooney (2024) |  |  |  |  |  |  |  |  |  |
| Sauter (2022) |  |  |  |  |  |  |  |  |  |
| Savoie (2023) |  |  |  |  |  |  |  |  |  |
| Sunesen (2015) |  |  |  |  |  |  |  |  |  |
| Tang (2015) |  |  |  |  |  |  |  |  |  |
| Taylor (2022) |  |  |  |  |  |  |  |  |  |
| Welzel (2011) |  |  |  |  |  |  |  |  |  |
| Yerrmailli (2020) |  |  |  |  |  |  |  |  |  |
| ^a^European Organisation for Research and Treatment of Cancer Quality of Life Questionnaire for use among patients with Colorectal Cancer- 29 checklist items  ^b^European Organisation for Research and Treatment of Cancer Quality of Life Questionnaire for use among patients with Colorectal Cancer- 38 checklist items  ^c^European Organisation for Research and Treatment of Cancer Quality of Life Questionnaire for use among patients with Anal Cancer- 27 checklist items  ^d^ Functional Assessment of Cancer Therapy- Colorectal  ^e^Female Sexual Function Index  ^f^Medical Outcomes Study Sexual Problems Scale  ^g^Patient Reported Outcomes Measurement Information System | | | | | | | | | |
